# Superhydrophobic WS_2_‐Nanosheet‐Wrapped Sponges for Underwater Detection of Tiny Vibration

**DOI:** 10.1002/advs.201700655

**Published:** 2018-01-26

**Authors:** Ruixin Xu, Kaili Zhang, Xiangyang Xu, Minghui He, Fachuang Lu, Bin Su

**Affiliations:** ^1^ School of Media and Communication Shenzhen Polytechnic Shenzhen 518055 China; ^2^ State Key Laboratory of Pulp & Paper Engineering South China University of Technology Guangzhou 510640 China; ^3^ Department of Chemical Engineering Monash University Clayton Vic 3800 Australia

**Keywords:** superhydrophobic materials, tungsten disulfide, underwater, vibration sensors

## Abstract

Underwater vibration detection is of great importance in personal safety, environmental protection, and military defense. Sealing layers are required in many underwater sensor architectures, leading to limited working‐life and reduced sensitivity. Here, a flexible, superhydrophobic, and conductive tungsten disulfide (WS_2_) nanosheets‐wrapped sponge (SCWS) is reported for the high‐sensitivity detection of tiny vibration from the water surfaces and from the grounds. When the SCWS is immersed in water, a continuous layer of bubbles forms on its surfaces, providing the sensor with two special abilities. One is sealing‐free feature due to the intrinsic water‐repellent property of SCWS. The other is functioning as a vibration‐sensitive medium to convert mechanical energy into electric signals through susceptible physical deformation of bubbles. Therefore, the SCWS can be used to precisely detect tiny vibration of water waves, and even sense those caused by human footsteps, demonstrating wide applications of this amphibious (water/ground) vibration sensor. Results of this study can initiate the exploration of superhydrophobic materials with elastic and conductive properties for underwater flexible electronic applications.

Vibration is derived from object motions, and quite common in our daily lives.^1^, ^2^ Accurate detection and monitoring of vibration play crucial roles in prewarning of infrastructure damage,^3^ real‐time health analysis,^4^, ^5^ environment protection,^6^ industrial manufacture,^7^ human recognition,^8^ and military defense.^9^ With the rapid development of exploiting marine resources, underwater vibration sensing attracts growing attentions.^10^, ^11^ Tremendous efforts have been made to fabricate diverse underwater vibration sensors, based on piezoelectric ceramics,^12^ fiber optics,^13^, ^14^ ultrasonic waves,^15^ or microelectromechanical systems (MEMSs).^16^ However, it has been extremely challenging for these techniques to attach the rigid sensing parts onto flexible clothing of swimmers/divers and prevent them from being corrosively attacked. Recent literatures^17^, ^18^, ^19^, ^20^ reported some wearable sensors with waterproof properties. In these designs, a close‐knit polymer sealing layer is prerequisite, raising two problems. The first one is their limited working life because of hard–soft material interfacial failure by incorporating rigid metal/semiconductor with soft elastomers, as well as polymeric aging issues.^21^ Water could permeate through the cracks of the interfaces or porosity of aged polymeric layers, and damage the device accordingly. The second one is their reduced sensitivity since the sealing layer physically restricts the responding spacing of key sensing parts. Therefore, new strategies to explore flexible, sealing‐free underwater vibration sensors are urgently required.

Superhydrophobic materials, bioinspired by natural creatures,^22^, ^23^, ^24^, ^25^ are a class of special substances that exhibit intrinsic water‐repellent capacity. In addition to fundamental research, these unique materials show promising practical applications in corrosion‐resistant coatings,^26^, ^27^ anti‐icing,^28^, ^29^ highly effective catalytic electrodes,^30^ crude‐oil spill treatments,^31^, ^32^ fighting against global‐warming,^33^ and other cutting across areas.^34^ Very recently, fabrications of stretchable/flexible superhydrophobic materials were reported,^26^, ^35^ implying the possibility of combining superhydrophobicity with elastic ability. In spite of numerous efforts have been made in the above‐mentioned studies, little has been reported on sensing underwater vibration by using these intrinsically water‐repellent materials to date.

Here we reported a flexible, superhydrophobic, and conductive tungsten disulfide (WS_2_) nanosheets‐wrapped sponge (SCWS) for the high‐sensitivity detection of tiny vibration not only from the water surface but also from the ground. The WS_2_ nanosheets uniformly wrapped the framework of the sponge, endowing the SCWS with elastic and conductive properties. Further coating of hydrophobic nanoparticles enabled the SCWS to become superhydrophobic. When the SCWS was immersed under the water, a continuous bubble layer formed on its surfaces. The bubbles were highly sensitive to even tiny pressure change caused by external vibration, and their physical deformations were quickly transferred into electrical signal through the flexible conductive sponge. As a result, the SCWS can be used to precisely detect tiny vibration of water waves, and even sense those caused by human footsteps, demonstrating wide application of this amphibious (water/ground) vibration sensor. The methodology reported here has great potential for developing new underwater vibration sensors, and opens up a new route to use superhydrophobic materials for a myriad of applications in future underwater flexible electronics.

A two‐step preparation procedure was used to fabricate the SCWS, as shown in **Figure** **1**a. Commercial melamine‐formaldehyde (MF) sponges were cut into 1 × 1 × 1 cm^3^ cubes, and used as the framework for WS_2_ nanosheet coating. Monolayer WS_2_ was used here due to their excellent mechanical stiffness, flexibility, and electrical carrier mobility.^36^, ^37^ The MF sponge cubes were immersed in the exfoliated WS_2_ nanosheet solution (the thickness and other details of WS_2_ nanosheets can be found in Figure S1 in the Supporting Information), then underwent vacuum degassing^38^ or centrifugation assistances,^32^ resulting in close contact of WS_2_ nanosheets with the sponge framework. As a result, the white sponge cubes became black (Figure S2, Supporting Information). Scanning electron microscopy (SEM) images show microscaled morphologies of sponges before (Figure 1b)/after (Figure 1c) the coating. WS_2_‐coated sponges remained their connected porous structures, implying that the coating procedure did not change the macrostructures of the sponge cubes. From magnified SEM images (Figure 1e,f), it was clearly found that the smooth MF framework was covered with a continuous layer of overlapped WS_2_ nanosheets. Following the increased amount of WS_2_ nanosheets from 1 to 14 wt% (Figure 1h), the electric conductivity of the sponge cubes was greatly improved by several orders of magnitude. In the meantime, hydrophobic nature^39^ of WS_2_ nanosheets changed the surface property of sponges, leading to their wettability transition from the Wenzel state (water contact angle (WCA) of ≈0°, Figure 1b) to semi‐Cassie state (WCA is around 145°, Figure 1c).

**Figure 1:**
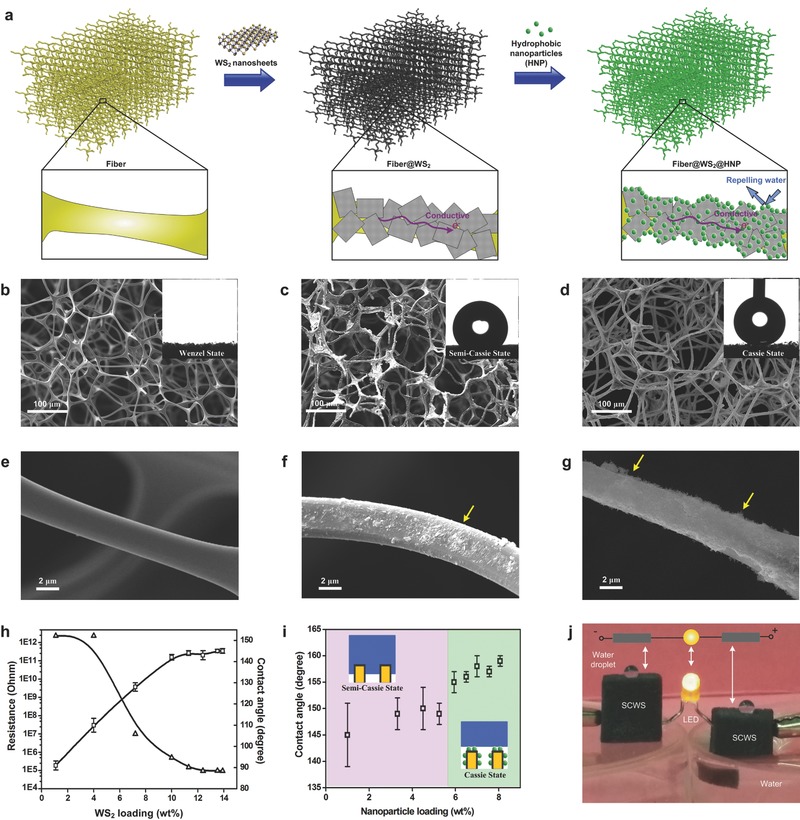
Fabrication of the SCWS cubes and their anti‐wetting and conductive properties. a) Schematic illustration of the fabrication of SCWS. Typical scanning electron microscopy (SEM) images of b) original melamine‐formaldehyde (MF), c) MF@WS_2_, and d) MF@WS_2_@hydrophobic‐nanoparticles (HNP) sponges. The inset images are optical images of a water droplet upon according sponge cubes. Panels (e–g) are magnified SEM images of panels (b–d), respectively. h) The dependence of electric resistance and water contact angle (WCA) of MF@WS_2_ sponges on the WS_2_‐loading weight. More WS_2_ nanosheets indicate lower electric resistances and higher WCAs. i) The dependence of WCA on the HNP‐loading weight. MF@WS_2_ sponges exhibited semi‐Cassie state hydrophobicity, and would be completely wetted after immersed in water. In contrast, MF@WS_2_@HNP (SCWS) sponges showed a Cassie state superhydrophobicity. j) The photograph of two SCWS cubes in lateral with an LED light, demonstrating their good superhydrophobic and electrical conductivity properties.

However, such semi‐Cassie state hydrophobic sponge cubes were quickly wetted after being immersed in water due to unstable solid–liquid–gas three phase contact line (Figure S3, Supporting Information). Thus, a further step of hydrophobic nanoparticle (HNP, the details of HNPs can be found in Figure S4**,** Supporting Information) coating was performed, shown in Figure 1a. Commercial HNPs not only improved the surface chemical composition but also contributed to considerable roughness to WS_2_‐nanosheet‐wrapped sponges (Figure 1d,g). Accordingly, SCWS with Cassie state superhydrophobicity (WCA > 155°, see Figure 1i) and a long durability > 30 d (Figure S5, Supporting Information) was prepared. Figure 1j shows a typical digital image of two pieces of the MF@WS_2_@HNP cubes. The spherical water drops on their tops, as well as irradiated light‐emitting diode (LED) lamp between them, indicate they simultaneously owned good superhydrophobic and electrical conductivity properties.

Owing to the elastic MF framework of the SCWS, it could be compressed to at least 85% strain and was able to almost recover to its original shape rapidly when the loading was removed (**Figure** **2**a–c). Therefore, this elastic SCWS cube can show a high‐sensitivity and stable response to external pressures through their physical deformation. The pressure sensitivity (*S*) of the SCWS has been tested by utilizing a computer‐controlled stepping motor and a force sensor, shown in Figure 2d. The sensitivity *S* can be defined as

(1) $$S=\frac{\frac{I-I_{\mathrm{off}}}{I_{\mathrm{off}}}}{\Delta P}$$

where *I* is the current when applied pressure on the devices, and *I*_off_ is the current of device with only base pressure, and Δ*P* is the change of applied pressure.

**Figure 2:**
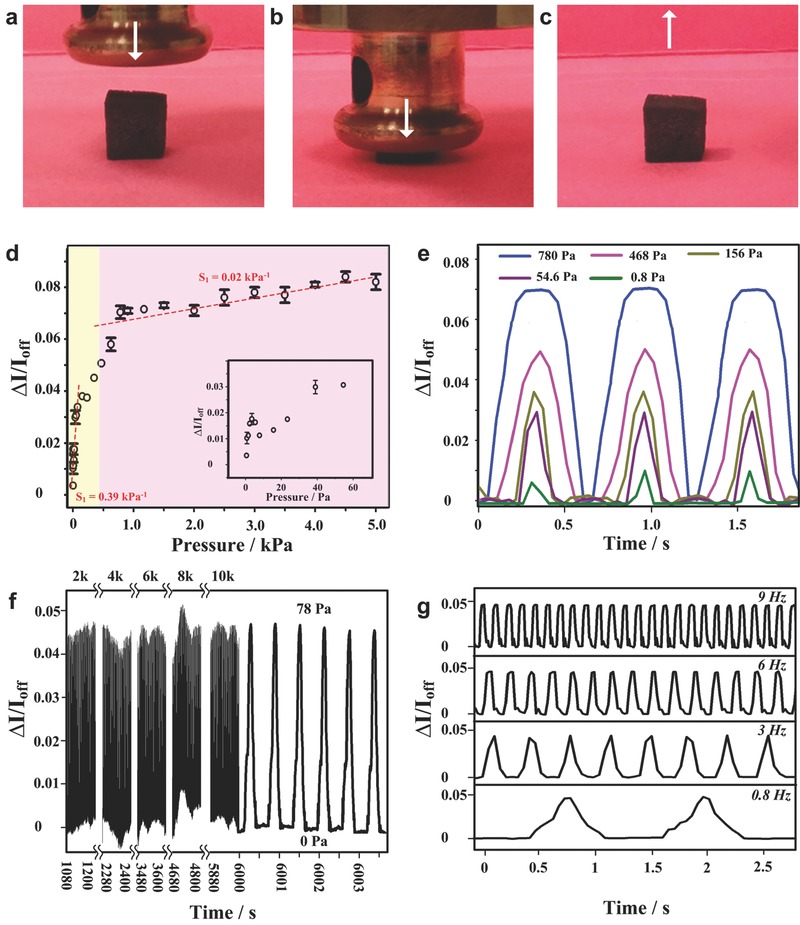
High‐sensitivity, stable and reliable flexible SCWS pressure sensors. Photographs of an SCWS cube a) before, b) being, and c) after pressed by a heavy matter. Because the framework of SCWS are porous MF elastomer, the cube could be physically compressed then recovered its appearance following the leaving of external pressure. d) Pressure‐response plots for the SCWS pressure sensor. The inset image is the plots in the low pressure range. e) Plots of current response of the sensor as a function of time (pressure input frequency: 1.6 Hz) for the applied pressures in the range of 0.8–780 Pa. f) The life‐time test under a pressure of 78 Pa at a frequency at 1.6 Hz. The current change curves were recorded after each 2000 cycles and 120 cycles of data were presented in each recording. g) Plots of current change of the SCWS as a function of time for diverse frequencies including 9, 6, 3, and 0.8 Hz. The relative humidity was 45% and the temperature was 25 °C.

The plots in Figure 2d can be divided into two regions due to the difference in sensitivity. In the low pressure region (yellow region, 0–125 Pa), the sensitivity of the device is 0.39 kPa^−1^, which is much higher than that of the relatively large pressure region (0.02 kPa^−1^ in pink region, 125–5000 Pa). The separated fittings of these points in both regions showed good linear behaviors (*R*_yellow_^2^ = 0.921, and *R*_pink_^2^ = 0.913), indicating the SCWS can serve as a reliable pressure sensor. The reason for different sensitivities can be explained based on different contact model of WS_2_ nanosheets. In low‐applied‐force yellow region, tiny pressure led to close contacts among the WS_2_ nanosheets wrapped along the MF fibers. Following the increase of external forces, the whole MF frameworks greatly deformed, yielding the further contact of WS_2_ nanosheets upon different MF fibers. However, the contact area tended to saturate in the pink region, and the applied pressure has little effect on the current variation. It should be noted that the sensitivity of SCWS was lower than some of the recently reported resistance type^40^, ^41^ or capacity type^42^, ^43^, ^44^, ^45^, ^46^, ^47^, ^48^ pressure sensors due to semiconductive nature of WS_2_ when compared with metallic counterparts.

The responses of our bioinspired sensors to dynamic mechanical pressures were characterized. To investigate the pressure range of the SCWS toward dynamic forces, a piezoelectric stepping positioner with minimum displacement of only 1 mm was applied to the sensors. As shown in Figure 2e, a pressure of 0.8 Pa could be detected, which indicates the weight of a water droplet (≈0.8 µL) on a surface of 10 mm^2^. At the higher pressure range (0.2–0.8 kPa), the error‐free, stable continuous responses could be observed. The cycling stability of the SCWS was tested under a pressure of 78 Pa at a frequency of 1.6 Hz (Figure 2f). The consistent resistance change with pressure applied on the surface of the pressure sensor can be maintained after 10 000 loading–unloading cycles. This result shows a strong adhesion between WS_2_ nanosheets and the MF foam, which guarantees this SCWS wtih a long working life and reliability. The response of the SCWS toward different frequencies was also investigated (Figure 2g). Note that the output electrical signals remained stable without obvious change in amplitude at typical frequencies of 0.8, 3.0, 6.0, and 9.0 Hz. The response time of SCWS under diverse frequencies has been carefully studied, as shown in Figure S6 in the Supporting Information. On the basis of these observations, the response time begun to delay when the frequency increased. Therefore, the sensor response seems to be reliable up to ≈10 Hz in terms of bandwidth. The response delay may be associated with the viscoelastic response of the MF supports.

This reliable SCWS pressure sensor was then used to detect vibration under the water surface. Two electric wires with bare ends were directly inserted into a piece of SCWS cube, allowing for the delivery of electrical signals from SCWS to the electrochemical workstation. To avoid physical separation between the wires and the SCWS during the testing, commercial glue was used to firmly anchor the electric wires onto the SCWS. Then, the SCWS‐based vibration sensor was immersed in a water sink with a depth of ≈5 mm (the distance between the water surface and the top of SCWS) through fixing above electric wires by commercial tapes (Figure S7a, Supporting Information). Different from dark appearance placed in the air (Figure 1j), shining silver‐mirror‐like surfaces existed around the SCWS (Figure S7b, Supporting Information). This result was due to the gas/liquid/solid composite interface^49^ in which a continuous gas bubble layer was trapped between the water and the SCWS surfaces.

The capacity of SCWS‐based vibration sensor was primarily investigated. We inserted a pair of tweezers into the water sink, which was ≈5 cm away from the sensor. Water waves could be generated by suddenly sharking the tweezers (**Figure** **3**a). The water waves continued to vibrate after lifting off the tweezers (Figure 3b), but their amplitude decreased due to energy dissipation resulted damping effect. The whole process was real‐time recorded by the SCWS sensor, as shown in Figure 3c. It is clear that the green resistance‐time curve remained stable at the beginning, and appeared several sharp downward peaks when the tweezers were inserted into the water. Then, alternative upward/downward peaks appeared due to cycled water waves generated by sharking the tweezers. The intensity of alternative peaks was gradually reduced following the attenuation of water waves, and returned to a stable line when the water surface stopped its motion. Notably, the SCWS failed to respond in certain time frames, which was attributed to its response limitation (<10 Hz, see Figure S6c in the Supporting Information). As a control experiment, we used MF@WS_2_ cube, which was in semi‐Cassie state and fully wetted after immersed in water (Figure S3, Supporting Information), as the sensor to monitor the similar process. Unfortunately, no obvious peak change existed (orange line in Figure 3c), indicating the importance of device wettability to dominate its sensing ability.

**Figure 3:**
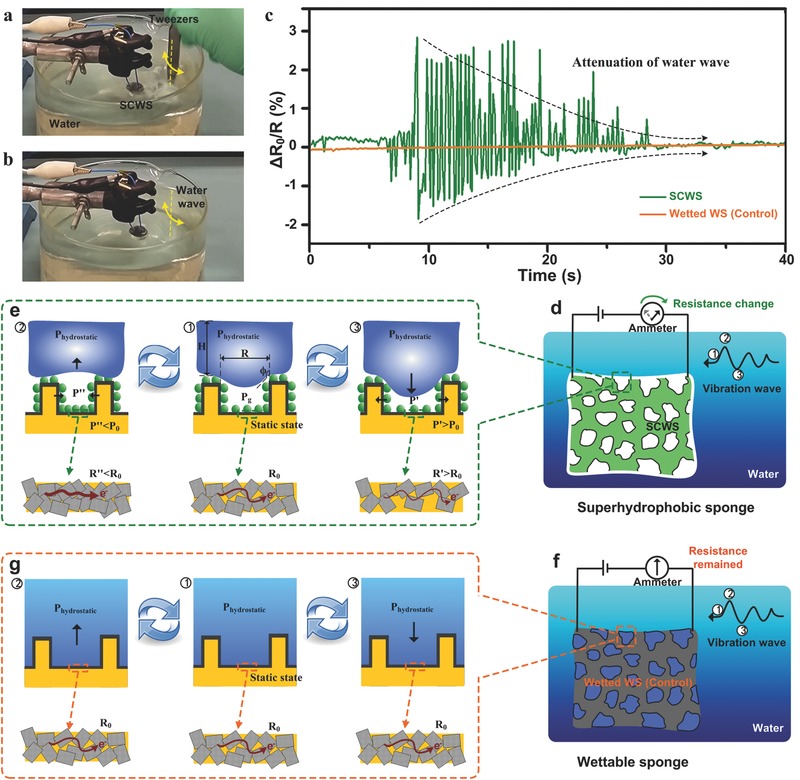
SCWS‐based underwater vibration sensors. a,b) Photographs of the SCWS sensor to respond the water waves. The SCWS sensors with input/output wires were immersed in a water tank (15 cm in diameter) with a depth of 5 cm. Continuous water waves were generated by suddenly sharking the tweezers inside the water. These mechanical energy was transferred to electrical signals by the SCWS, and recorded by a connected electrochemical workstation. c) The electric resistance change of SCWS (green line) and wetted MF@WS_2_ (orange line) sensors as a function of time. When suffering the similar water waves generated by sharking the tweezers, anti‐wetting SCWS sensor can monitor the whole process by upward/downward peaks while wetted MF@WS_2_ showed negligible response. The applied voltage in all the electrical tests was 0.5 V, and the temperature was ≈20 °C. Schematic illustration of d) the SCWS and f) wetting WS sensor meeting with vibration under the water. e) Schematic illustrations of magnified images showing the solid–liquid–gas three phase contact line of the SCWS under the water. In a static state (①), a concave meniscus existed due to considerable hydrostatic pressure. Following an upward vibrating wave, the trapped bubbles would expand (②), leading to reduced inside gas pressure and consequent compressing of sponge. Therefore, WS_2_ nanosheets were compressed to contact more close, yielding a reduced resistance for the SCWS. In contrast, downward vibration wave led to compressed gas bubbles (③), indicating partly separated WS_2_ nanosheets and corresponding resistance increase. g) Schematic illustrations of magnified images showing the solid–liquid two phase contact line of the MF@WS_2_ under the water. No matter in a static state (①) or suffering an upward (②)/downward (③) vibrating wave, WS_2_ nanosheets upon the MF framework remained stable, showing negligible electric signals, showing the crucial role of the bubbles contributed by superhydrophobic SCWSs.

As we described in the “Experimental Section,” the electric wires were physically inserted in the SCWS and closely sealed by the commercial glue. This treatment can prevent the detachment of electric wires to the SCWS when suffering the vibration, which has been confirmed by the control experiment (orange line in Figure 3c). The MF@WS_2_ cube showed negligible response to the applied vibration, indicating the close attachment between the electric wires and SCWS.

From the result comparison between Cassie‐ and semi‐Cassie stated sponge sensors (Figure 3c), it is obvious that the air bubbles trapped between the water and sponge surfaces play a crucial role in sensing the water vibration (Figure 3d). The pressure of the captive air bubble, *P*_g_, can be calculated according to the following equation^50^

(2) $$P_{g}=P_{0}+\rho \cdot g\cdot H-\frac{2\gamma \cdot \cos\phi }{R}$$

where *P*_0_ is the standard atmosphere pressure, ρ is the density of water, *g* is the gravitational acceleration, *H* is the depth below the water surface, γ is the water–air interface surface tension, φ is the equilibrium contact angle, *R* is the diameter of the bubbles, and *H*, φ, and *R* are the geometries depicted in the middle part of Figure 3e.

From the above equation, it is easy to find that the *P*_g_ is commonly larger than the hydrostatic pressure at the same depth because the φ is >90°. When the water surface was quiet, a continuous layer of air bubbles existed upon the SCWS surfaces (Figure S7c, Supporting Information). Being disturbed by a pulse of water wave, the bubbles would physically deform. Taking the upward vibration wave for an example (left part in Figure 3e), the bubbles trapped between the water and the sponge would expand, leading to reduced P_g_ according to ideal gas equation of state

(3) P⋅V=n⋅R⋅T

where *P* is the gas pressure, V is the volume of gas, n is the number of moles of gas, R is the ideal gas constant, and T is the absolute temperature.

Therefore, the expanding of the gas bubbles (increased *V*) decreased the inside gas pressure to P″ when assuming the temperature (*T*) was a constant. Reduced gas pressure would lead to the compressing of sponge framework (the elastic property of SCWS can be found in Figure 2a–c), yielding closer contact among the WS_2_ nanosheets. Thus, the resistance of sensor was reduced, showing a downward peak in its vibration responding line (Figure 3e). In contrast, downward vibration wave led to compressed gas bubbles, indicating increased gas pressure to stretch the sponge framework. As a result, the WS_2_ nanosheets were partly separated, resulting larger resistance, and the upward responding peak (Figure 3d). Following cycled vibrating of water waves, the air bubbles trapped between water and sponge surfaces were alternatively compressed/stretched, allowing for high‐sensitivity detection of tiny water waves. For fully wetted MF@WS_2_ sensor, air bubbles disappeared under the water surface. The porous sponge framework was filled with water, indicating the vibration waves have to directly contract with the WS_2_‐wrapped framework (Figure 3f,g). However, the tiny water waves were not strong enough to make the deformation of MF@WS_2_ sensor, leaving a stable line (Figure 3c). Underwater pressure sensitivity of the SCWS was tested, as shown in Figure S8 in the Supporting Information. Different from that at the atmosphere (Figure 2d), the linear dependence of electrical response of the device on the pressure was greatly reduced, which was caused by the existence of air bubbles between the SCWS and the water.

For a systematic study of detecting water waves by the SCWS sensor, regular water waves were generated by beating the water surface through a home‐made setup (**Figure** **4**a). A speed‐controllable motor in lateral with a plastic plate was used to provide water‐beating behaviors, allowing for the generation of tunable water waves. The dependence of resistance change of SCWS sensor on responding distance has been investigated, as shown in Figure 4b. The responding distance was defined as the length between the vibration resource and the sensor. Longer transport distance would dissipate the mechanical energy of water waves, leading to weaker electrical signals. Thus, the sensor resistance decreased from 6.2% to 0.7% when increasing the distance from 2.5 to 10 cm. The minimum detectable vibration amplitude of the SCWS was ≈1 mm and the frequency should be lower than 10 Hz. The stability of the sensor has been tested by repeatable beating of water waves by the plastic plate, as shown in Figure 4c. Error‐free, stable, and repeatable responses of SCWS sensor appeared when stimulated by similar water waves, indicating our SCWS can serve as a reliable underwater vibration sensor.

**Figure 4:**
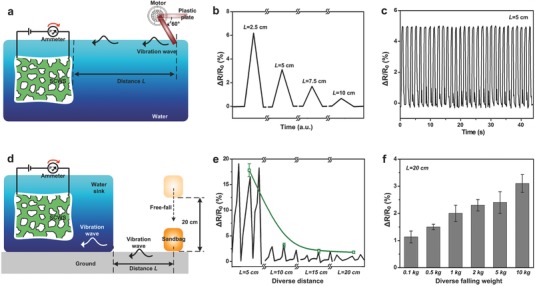
Detection of regular water waves and vibration from the ground. a) Schematic illustration of the SCWS sensor to detect regular water waves. A home‐made speed‐controlled motor in lateral with a plastic plate was used to provide regular vibration sources. b) The electric resistance change of SCWS sensor as a function of the distance between the sensor and the vibration source. c) Repeatable electric responding of SCWS sensor to the same stimuli. SCWS sensor can show error‐free, stable, and continuous responses to repetitive water waves, indicating its reliability as the vibration sensor. The distance between the sensor and the vibration source was fixed at 5 cm. d) Schematic illustration of the SCWS sensor to detect vibration waves from the ground. To generate controllable vibration from the ground, sandbags with diverse weights were dropped from a 20 cm height. e) The electric resistance change of SCWS sensor as a function of the distance between the sensor and the vibration source. Closer distance led to stronger signals. f) The electric resistance change of SCWS sensor as a function of the weight of sandbags. The distance between the sensor and the vibration source was fixed at 20 cm. The applied voltage in all the electrical tests was 0.5 V, and the temperature was ≈20 °C.

Besides working under the water, unexpected sensing ability of SCWS toward vibration from the ground has been studied. The SCWS sensor was immersed in the center of the water sink (the distance between the sensor and the sink wall was ≈7 cm), and horizontally placed on the ground (Figure 4d). To generate controllable vibration from the ground, sandbags with diverse weights were dropped from a 20 cm height. The free‐falling of the sandbag transferred its gravitational potential energy to vibration‐type mechanical energy. The vibration waves transported through the ground, met the sink wall then changed to water waves, and were finally detected by the SCWS sensor. Figure 4e shows the dependence of sensor resistance change on the distance of sandbags away from the sink wall. The mechanical vibrating energy was gradually dissipated along the transporting route. Thus, the sensor resistance decreased from 17.9% to 1.1% when increasing the distance from 5 to 20 cm. Besides changing the vibrating distance, the intensity of vibration waves was tuned by increasing the weight of sandbags, as shown in Figure 4f. When the weight of sandbags increased from 0.1 to 10 kg, stronger vibration waves could be generated. As a result, the signals of SCWS sensor were raised three times of the magnitude.

Apart from sandbags, human footsteps can also be monitored by the SCWS sensor. When the volunteer planned to walk, he would first lift one foot away the ground, allowing for increased pressure from his body toward the ground (**Figure** **5**a). The pressure change led to a tiny vibration, which was monitored, as shown in Figure 5b. The distance between the volunteer and the sensor was ≈100 cm and the weight of volunteer is around 70 kg. When one of his feet was raised, the resistance of sensor changed 4.1% accordingly. The responding time is ≈0.125 s. To prove the repeatability of this monitoring, the dependence of the resistance change on the rotation angle was recorded and observed to demonstrate the high‐sensitivity of SCWS sensor (Figure 5c). Eight directions, with three distances between the sensor and the volunteer, including 20, 50, and 100 cm, were tested. Following the decrease of sensing distance, the electrical signal increased from 4.2% to 10.1%, showing its high‐sensitivity toward the foot lifting and reliability of the SCWS sensor. Walking is alternative lifting of one foot while keeping another contacting with the ground, and there is a period of double support.^51^ Therefore, the vibration waves on the ground can be continuously generated, and monitored by the SCWS sensor (Figure 5d). It is clearly shown that the electrical signal increased when the volunteer walked toward the sensor. Therefore, our SCWS sensor showed its amphibious (water/ground) sensing abilities toward tiny water waves, and even detection of human footsteps.

**Figure 5:**
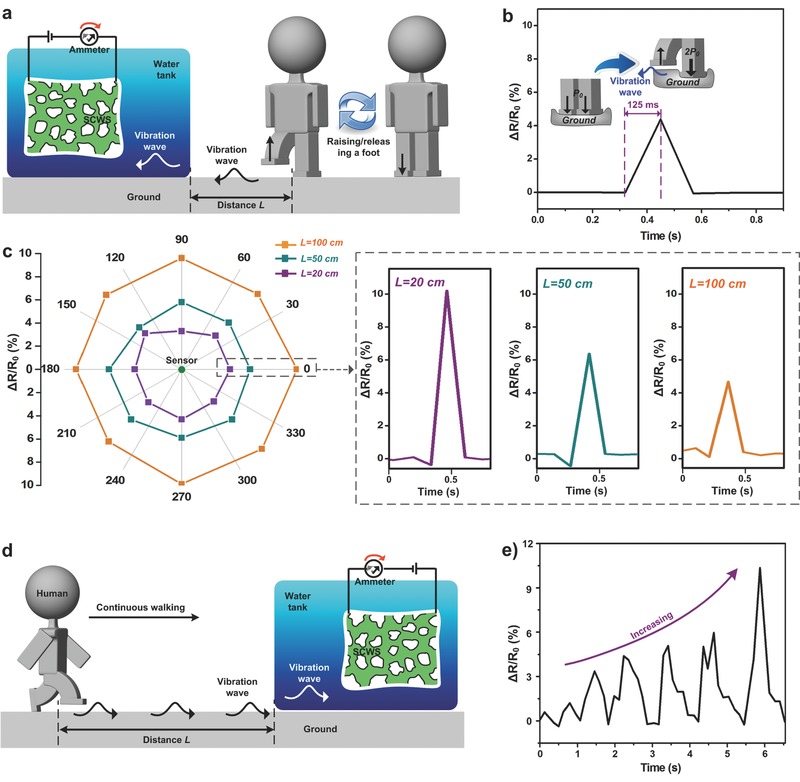
Detection of human footsteps on the ground. a) Schematic illustration of the SCWS sensor to detect the lifting of one foot away from the ground by the volunteer. When the volunteer lifted his one foot, the pressure from human toward the ground would be increased, resulting a tiny vibration. b) The electric resistance change of SCWS sensor as a function of time. The pressure change on the ground by lifting foot has been recorded by the SCWS sensor. c) The dependence of the resistance change on the rotation angle by lifting one foot of the volunteer. Three distances have been tested as 20 cm (purple dots), 50 cm (green dots), and 100 cm (orange dots), respectively. The right part are one group of representative resistance change on the time. d) Schematic illustration of the SCWS sensor to detect the footsteps of the volunteer. e) The electric resistance change of SCWS sensor as a function of time. The electrical signal increased when the volunteer walked toward the sensor. The applied voltage in all the electrical tests was 0.5 V, and the temperature was ≈20 °C.

Different from traditional underwater vibration sensors based on piezoelectric ceramics,^12^ fiber optics,^13^, ^14^ ultrasonic waves,^15^ or MEMSs,^16^ or flexible wearable sensors,^17^, ^18^, ^19^, ^20^ the key functional unit in our SCWS sensor is the air bubbles trapped between the water and the surfaces of a superhydrophobic sponge. The physical deformations of the trapped air bubbles played a crucial role in transferring mechanical energy into readable signal (Figure 3d,e). The lifetime of the bubbles depends on the diffusion of gas into the water.^52^ To solve this gas‐diffusing problem, it is easy to regenerate the bubbles by air‐blowing the SCWS sensor under the water, or just directly lifting the sensor to the atmosphere then immersing it again under the water surface. In these cases, the air bubble layer would be regenerated and continue to work.

It should be aware of that the SCWS sensor technology reported here is at its infant stage. There are still some aspects to be improved. For instances, the functional part, superhydrophobic SCWS cube was connected by electric wires where sealing coatings were required. The polymer aging issue^21^ would exist here and especially for the joint parts between the wires and the SCWS cubes. Therefore, the next step for improving the SCWS sensor will logically be wireless connection by placing the battery and wireless transmitters inside its body, so that the whole sensor will be intrinsic anti‐wetting and free of underwater polymeric aging issues.

As Albert Einstein said: “everything in life is vibration.” Detection and monitoring tiny vibration is of great importance in practical applications.^3^, ^4^, ^5^, ^6^, ^7^, ^8^, ^9^, ^10^, ^11^ In this study, we have demonstrated the ability of the SCWS sensor to convert mechanical energy changes to electric signals and its interesting application to detect vibration not only from the water but also those from the ground near the water sink. The SCWS sensor was able to detect the motion of a 70 kg volunteer who was just lifting his one foot away from the ground or continuously walking, indicating wide applications of this amphibious (water/ground) vibration sensor. Such a superhydrophobic sensor design will provide a new strategy for high‐sensitivity monitoring of tiny vibrations.

## Experimental Section

*Fabrication of SCWS Cubes*: Commercial melamine–formaldehyde sponges were cut into 1 × 1 × 1 cm^3^ cubes, then cleaned by alternative acetone and distilled water for two times in an ultrasonic cleaner. The resulted sponge cubes were dried in a vacuum oven at 100°C for 2 h to completely remove potential moisture. Then, these precleaned sponge cubes were dipped into a dispersion of WS_2_ nanosheets in ethanol (0.1 wt%, purchased from Nanjing Muke Nano company), then underwent vacuum degassing at 100 °C for 2 h or centrifugation assistances at ≈1000 rpm for 1 min , allowing for close contact of WS_2_ nanosheets with the sponge framework. The amount of WS_2_ nanosheets was tunable by repeating the “dipping and drying” process. For further hydrophobic modification, WS_2_‐wrapped sponges were dipped into a dispersion of commercial hydrophobic fumed silica nanoparticles (Aerosil R202, average particle size 14 nm, Evonik Degussa Co.) in ethanol (3 wt%), then underwent vacuum degassing at 100°C for 2 h or centrifugation assistances at ≈1000 rpm for 1 min, yielding SCWS cubes.

*Sensor Fabrication*: Two electric wires with bare ends were directly inserted into a piece of SCWS cube, allowing for the delivery of electrical signals from SCWS to the electrochemical workstation. To avoid physical separation between the wires and the SCWS during the testing, commercial sealing glue was used to firmly anchor the electric wires onto the SCWS.

*Characterizations*: The structures of SCWS cubes and control samples were examined using a scanning electron microscope (JEOL, JSM‐6700F, Japan) with an accelerating voltage of 3.0 kV. Static WCAs were measured on a DataPhysics OCA20 contact angle system at ambient temperature. The average WCA was obtained by measuring more than five different positions on the same sample. Optical images and photos were recorded by using a commercial 7.2 megapixel digital camera (Sony, DSC‐W120, Japan).

*Test of Sensor Behaviors*: The SCWS sensors with input/output wires were immersed in a water tank (15 cm in diameter) with a depth of 5 cm. The electrical characteristics for all the vibration monitoring were recorded by the Parstat 2273 Electrochemical System (Princeton Applied Research). A home‐made speed‐controlled motor in lateral with a plastic plate was used to provide regular vibration sources. To avoid false signals generated from other vibration sources, including human walking around, noises or working vacuum pumps, all the testing were performed at night where only the tester and the volunteer appeared.

## Conflict of Interest

The authors declare no conflict of interest.

## Supporting information

Supplementary
