# Supplementary material for: Superhydrophobic WS2‐Nanosheet‐Wrapped Sponges for Underwater Detection of Tiny Vibration
Source: Adv Sci (Weinh). 2018 Jan 26;5(4):1700655. doi: 10.1002/advs.201700655 (PMC5908356; doi:10.1002/advs.201700655)
Supplement: Supplementary file 1 — Supplementary [file ADVS-5-1700655-s001.pdf]

Copyright WILEY-VCH Verlag GmbH & Co. KGaA, 69469 Weinheim, Germany, 2018.

ADVANCED SCIENCE
Open Access

Supporting Information

for *Adv. Sci.*, DOI: 10.1002/advs.201700655

Superhydrophobic WS_2-Nanosheet-Wrapped Sponges for Underwater Detection of Tiny Vibration

Ruixin Xu, Kaili Zhang, Xiangyang Xu, Minghui He, Fachuang Lu, and Bin Su**

Copyright WILEY-VCH Verlag GmbH & Co. KGaA, 69469 Weinheim, Germany, 2016.

Supporting Information

Superhydrophobic WS_2-nanosheet-wrapped sponges for underwater detection of tiny vibration

Ruixin Xu[†1,2], *Kaili Zhang*[†2], *Xiangyang Xu*[1], *Minghui He*[*2], *Fachuang Lu*[2], *Bin Su*[*3]

[1]School of Media and Communication, Shenzhen Polytechnic, Shenzhen, 518055, China

[2]State Key Laboratory of Pulp & Paper Engineering, South China University of Technology, Guangzhou 510640, China. E-mail: heminghui_2008@163.com

[3]Department of Chemical Engineering, Clayton, Vic 3800, Australia. E-mail: subin0000@iccas.ac.cn

[†]These authors contributed equally to this work

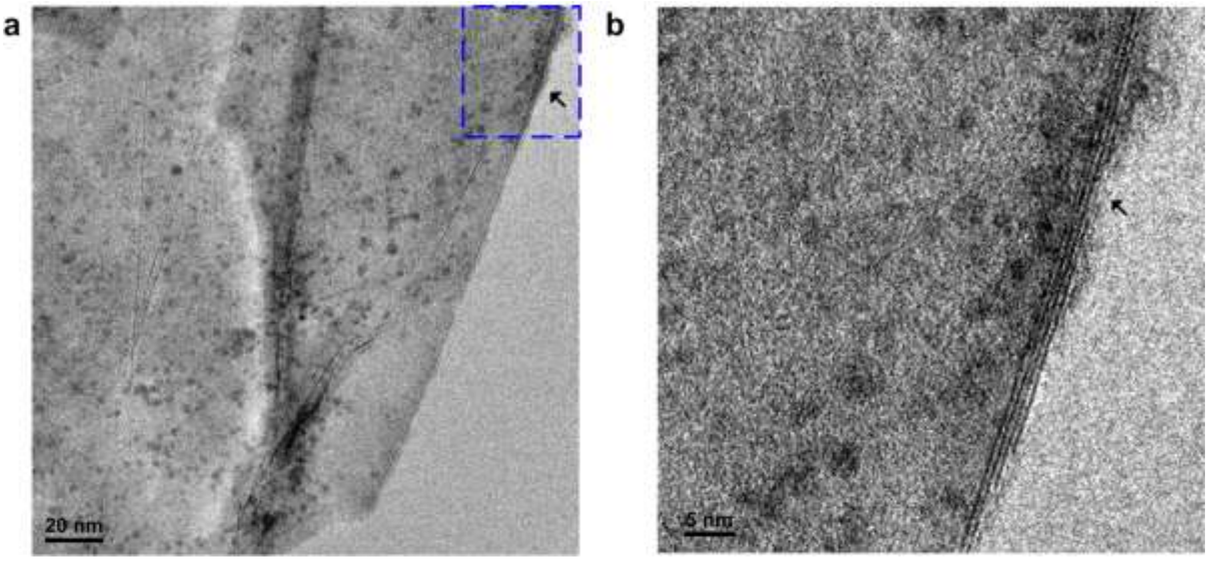

Supplementary Figure 1. Transmission electron micrograph (TEM) of exfoliated tungsten disulfide (WS_2) nanosheets. (**b**) is magnified image of (**a**), showing the WS_2 nanosheets are few atomic layers.

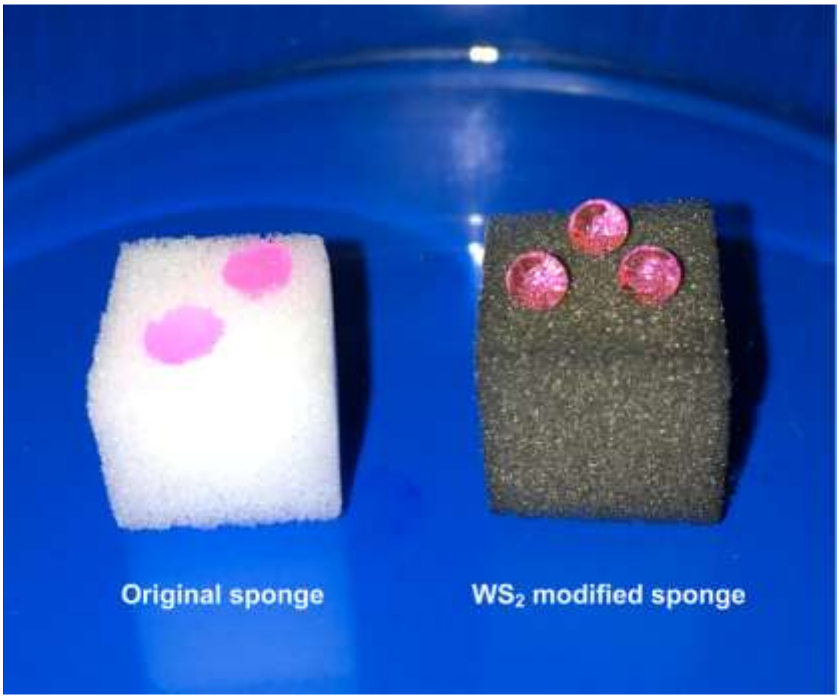

Supplementary Figure 2. Photograph of commercial melamine-formaldehyde (MF) sponges before/after WS_2 nanosheets modification. The MF sponge cubes were immersed in the exfoliated WS_2 nanosheet solution, allowing for close contact of WS_2 nanosheets with the sponge framework. As a result, the white hydrophilic sponge cubes (left) became black and hydrophobic (right). The water droplets were dyed for easy observation.

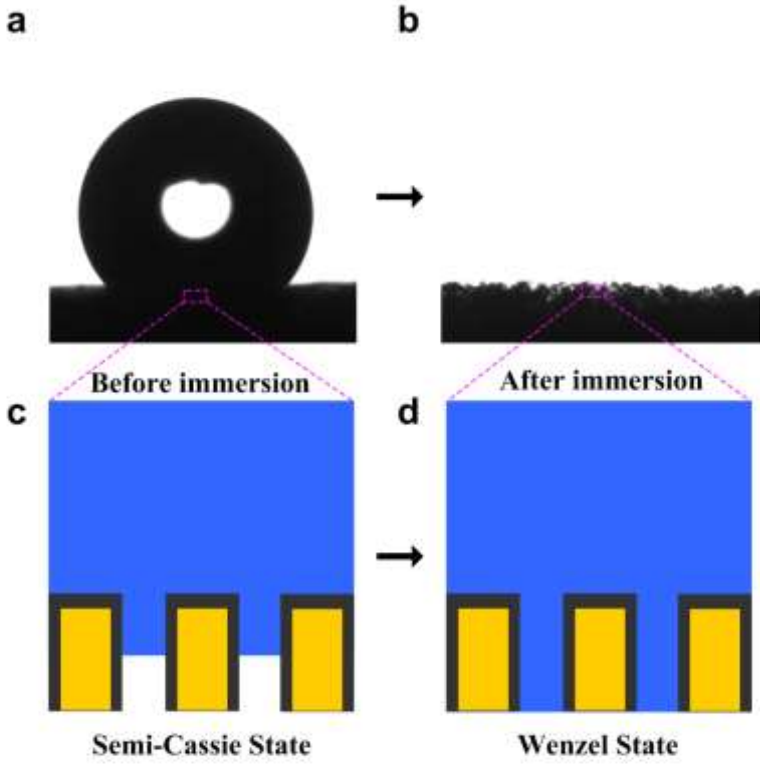

Supplementary Figure 3. Semi-Cassie state hydrophobic WS_2 wrapped sponge cubes were quickly wetted after immersed in water. Optical images of a water droplet upon WS_2 wrapped sponge cubes (**a**) before and (**b**) after immersion in the water. (**c,d**) are schematic illustrations of solid-liquid-gas three phase contact lines of (**a,b**), respectively. Unstable semi-Cassie state was changed to completely wetting Wenzel state after immersion in the water.

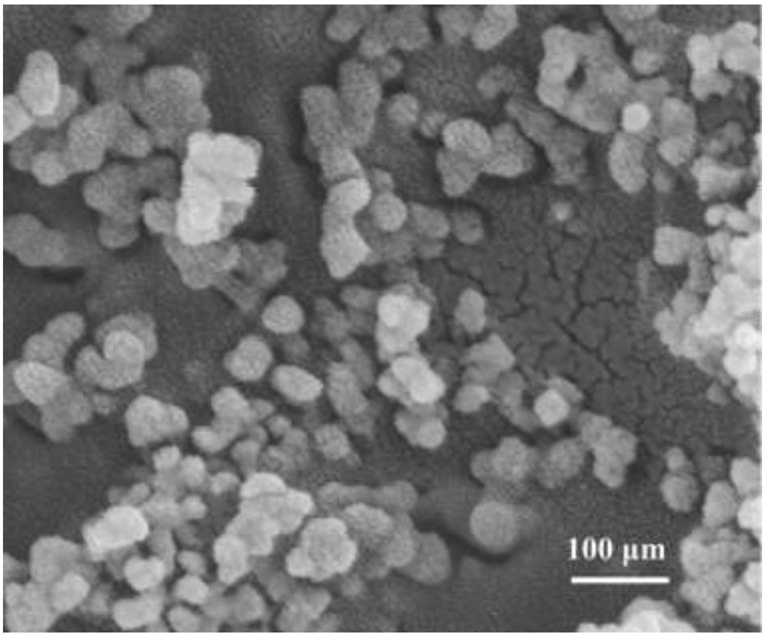

Supplementary Figure 4. SEM of commercial hydrophobic fumed silica nanoparticles (HNPs). The average size of HNPs was ~14nm. Several particles would aggregate, yielding a larger appearance in the SEM observation.

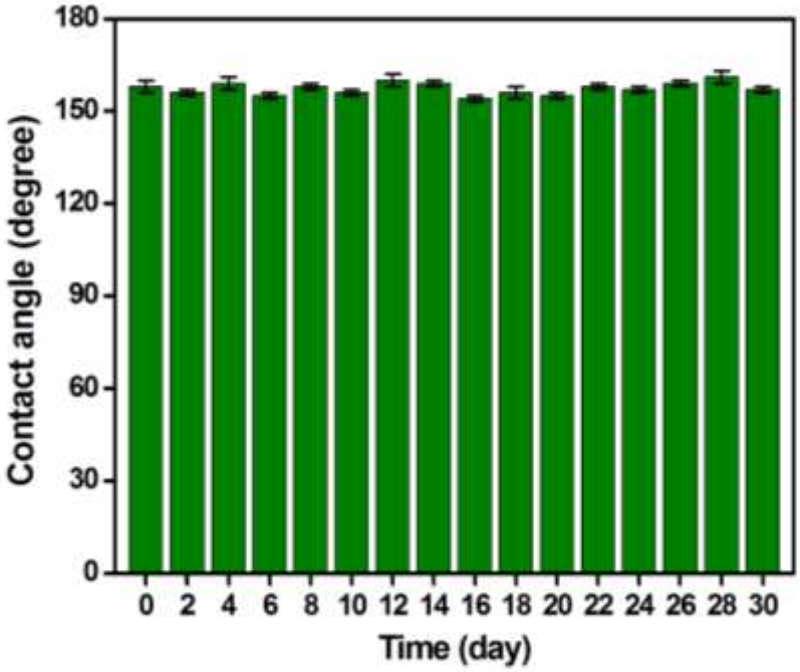

Supplementary Figure 5. Stable superhydrophobicity of MF@WS_2@HNP sponge cubes. The water contact angle (WCA) of modified sponges as a function of time. Owing to the coating of HNPs, as-prepared sponge cubes showed a long durability of anti-wetting ability with a WCA >155° for more than 30 days.

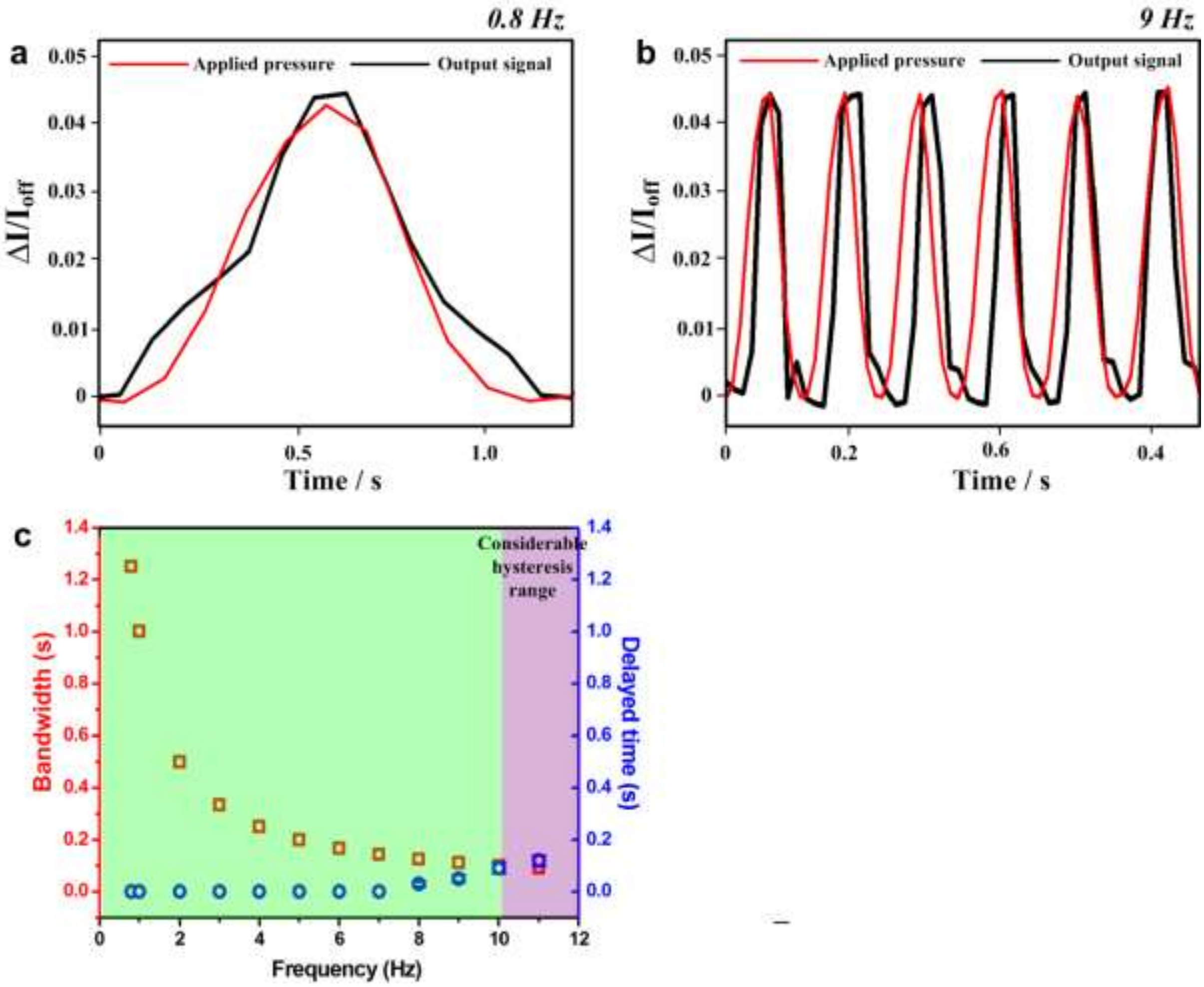

Supplementary Figure 6. Time-resolved measurements of the SCWS sensor response. Representative time-resolved plots of the output signal for an applied pressure frequency of **a**) 0.8 Hz and **b**) 4.17 Hz. The applied pressures were 78 Pa. **c**) The dependence of bandwidth (red plots) and sensor hysteresis time (blue plots) on the frequency. The hysteresis time increased from 0 s to nearly 0.12 s following the increase of frequency.

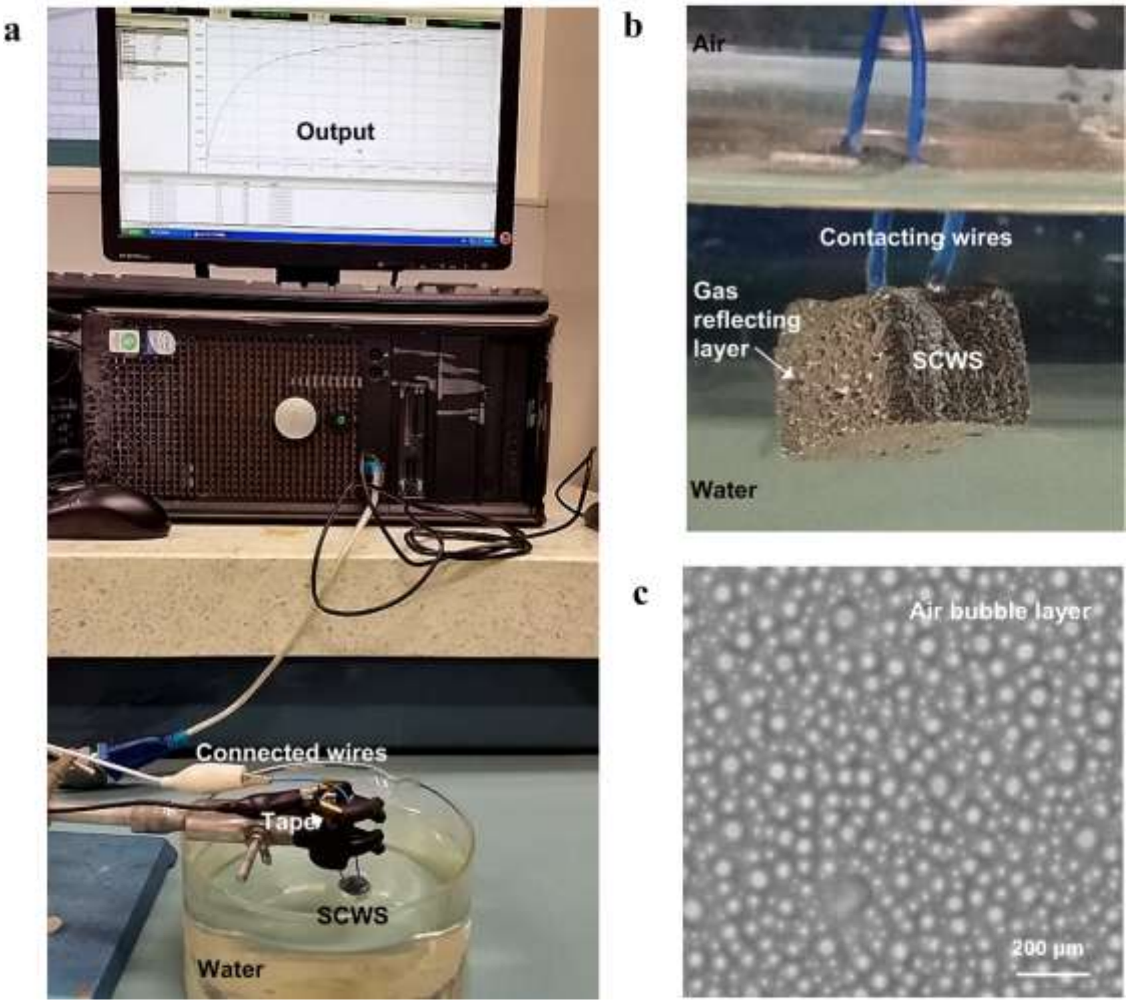

Supplementary Figure 7. a) Photograph of the setup of underwater vibration sensor based on WS_2 nanosheets wrapped sponges (SCWSs). Two electric wires with bare ends were directly inserted into a piece of SCWS cube, allowing for the delivery of electrical signals from SCWS (bottom part) to the electrochemical workstation (top part). The SCWS based vibration sensor was immersed in a water sink with a depth of ~5 mm through fixing the electric wires by commercial tapes. The applied voltage in all the electrical tests was 0.5 V, and the temperature was ~20 °C. **b**) The photograph of one SCWS sensor immersed under the water. Shining silver-mirror-like surfaces exist around the SCWS, indicating the existence of a layer of air bubbles trapped between the water and the SCWS surfaces. **c**) Photograph of underwater bubble layers upon the SCWS cube.

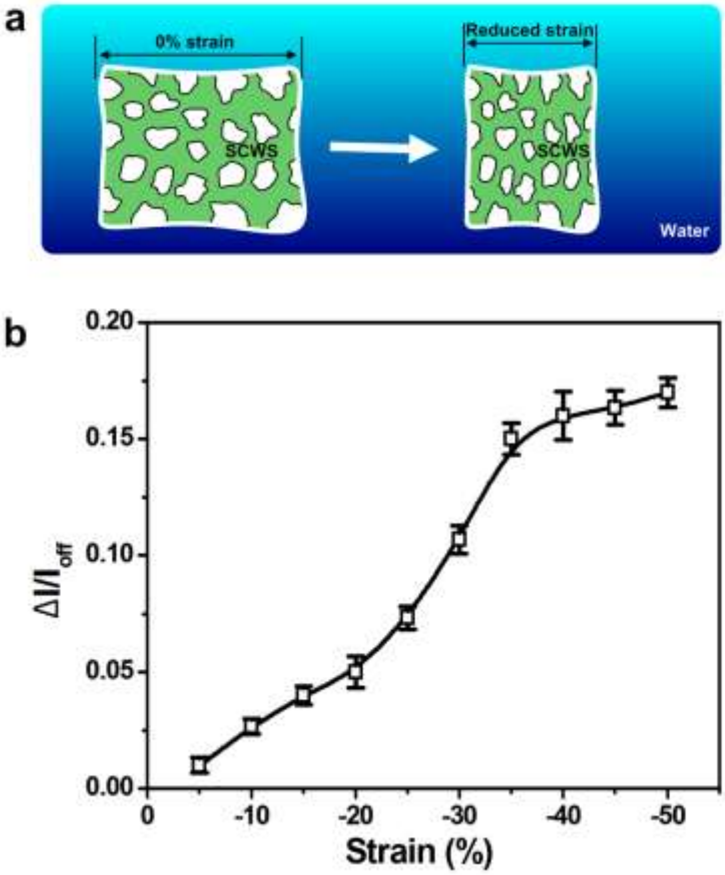

Supplementary Figure 8. Underwater response of the SCWS pressure sensor. **a**) Schematic illustration of the SCWS sensor with controlled strains. A home-made clip was used to fix the SCWS in a certain strain. **b**) Strain-response plots for the SCWS pressure sensor under the water. The linear dependence of electrical response of the device on the pressure was greatly reduced, which was caused by the existence of air bubbles between the SCWS and the water.
